# Ethyl 3′-(2,4-dichloro­phen­yl)-5′-hydr­oxy-5′-methyl-4′,5′-dihydro­spiro­[fluorene-9,2′(3′*H*)-furan]-4′-carboxyl­ate

**DOI:** 10.1107/S1600536809011854

**Published:** 2009-04-08

**Authors:** M. NizamMohideen, S. Thenmozhi, A. SubbiahPandi, G. Savitha, P. T. Perumal

**Affiliations:** a Department of Physics, The New College (Autonomous), Chennai 600 014, India; b Department of Physics, Presidency College (Autonomous), Chennai 600 005, India; c Organic Chemistry Division, Central Leather Research Institute, Chennai 600 020, India

## Abstract

The furan ring and the five-membered fluorene unit in the title compound, C_26_H_22_Cl_2_O_4_, adopt envelope conformations. Inter­molecular C—H⋯O inter­actions between symmetry-related mol­ecules involving two C—H groups and an O atom as a bifurcated acceptor generate centrosymmetric hydrogen-bonded dimers with cyclic *R*_2_^2^(16) and *R*_2_^2^(8) ring motifs. A short C—H⋯Cl intramolecular contact occurs in the molecule.

## Related literature

For spiro compounds in pharmacologically active alkaloids, see: Cravotto *et al.* (2001 ▶). For the anticonvulsant activity of fluorene derivatives, see: Vanvakides *et al.* (2004 ▶). Fluorene derivatives, including polyfluorenes and oligofluorenes, are promising candidates for blue light-emitting materials in organic light-emitting devices (Muller *et al.*, 2003 ▶), organic phototransistors (Saragi *et al.*, 2004 ▶), non-linear optics (Kim *et al.*, 1998 ▶) and photochromic materials (Chun *et al.*, 2003 ▶). For the biological activity of furan derivatives and annulated furan derivatives and their use as precursors for the synthesis of natural products, see: Greve & Friedrichsen (2000 ▶). For hydrogen-bond motifs and ring puckering parameters, see: Bernstein *et al.* (1995 ▶); Cremer & Pople (1975 ▶); Nardelli (1983 ▶). For a related spiro-linked system, see: Feng *et al.* (2004 ▶).
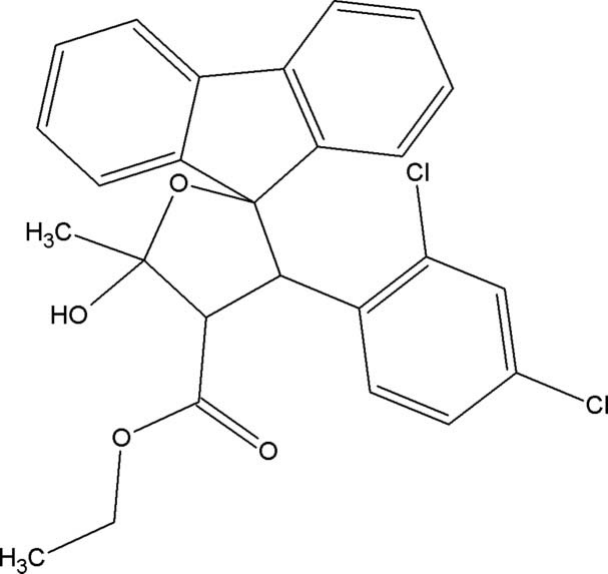


## Experimental

### 

#### Crystal data

C_26_H_22_Cl_2_O_4_
*M* *_r_* = 469.34
Monoclinic, 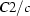

*a* = 28.6811 (13) Å
*b* = 9.0600 (4) Å
*c* = 17.4074 (8) Å
β = 92.072 (3)°
*V* = 4520.4 (4) Å^3^
*Z* = 8
Mo *K*α radiation
μ = 0.32 mm^−1^
*T* = 293 K
0.25 × 0.20 × 0.20 mm

#### Data collection

Bruker Kappa APEXII CCD diffractometer
Absorption correction: multi-scan (*SADABS*; Bruker 2004 ▶) *T*_min_ = 0.916, *T*_max_ = 0.938
22504 measured reflections
5338 independent reflections
3663 reflections with *I* > 2σ(*I*)
*R*_int_ = 0.056

#### Refinement

*R*[*F* ^2^ > 2σ(*F* ^2^)] = 0.048
*wR*(*F* ^2^) = 0.151
*S* = 1.04
5338 reflections
291 parameters
H-atom parameters constrained
Δρ_max_ = 0.38 e Å^−3^
Δρ_min_ = −0.35 e Å^−3^

### 

Data collection: *APEX2* (Bruker, 2004 ▶); cell refinement: *SAINT* (Bruker, 2004 ▶); data reduction: *SAINT*; program(s) used to solve structure: *SHELXS97* (Sheldrick, 2008 ▶); program(s) used to refine structure: *SHELXL97* (Sheldrick, 2008 ▶); molecular graphics: *ORTEP-3* (Farrugia, 1997 ▶); software used to prepare material for publication: *SHELXL97* and *PLATON* (Spek, 2009 ▶).

## Supplementary Material

Crystal structure: contains datablocks global, I. DOI: 10.1107/S1600536809011854/fl2242sup1.cif

Structure factors: contains datablocks I. DOI: 10.1107/S1600536809011854/fl2242Isup2.hkl

Additional supplementary materials:  crystallographic information; 3D view; checkCIF report

## Figures and Tables

**Table 1:** Hydrogen-bond geometry (Å, °)

*D*—H⋯*A*	*D*—H	H⋯*A*	*D*⋯*A*	*D*—H⋯*A*
C2—H2*A*⋯O3^i^	0.98	2.37	3.331 (3)	167
C6—H6⋯O3^i^	0.93	2.55	3.393 (3)	151
C3—H3⋯Cl1	0.98	2.57	3.082 (2)	113

Symmetry codes: (i) 
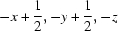
.

**Fig. 1.**
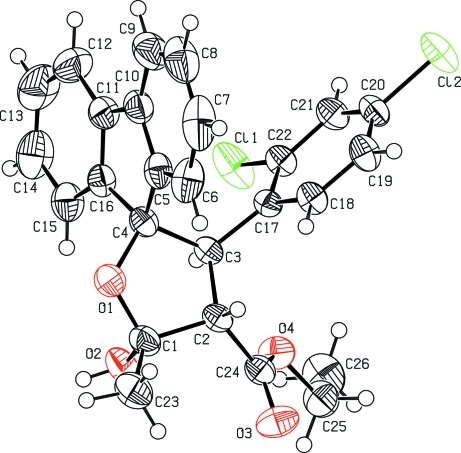
The molecular structure of (I), showing the atom-numbering scheme for. Displacement ellipsoids are drawn at the 50% probability level.

**Fig. 2.**
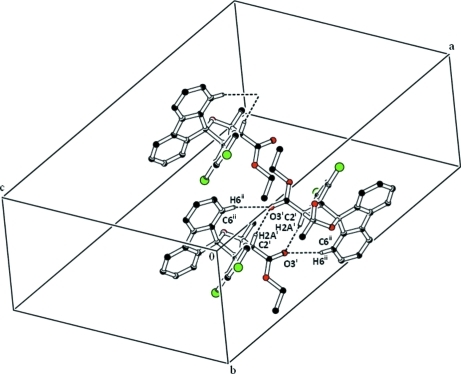
The crystal packing of compound (I), showing the R22(16) and R22(8) rings. Hydrogen bonding is shown as dashed lines. H atoms not involved in the hydrogen bonding have been omitted for clarity. [Symmetry codes: -x + 1/2, -y + 1/2, -z]

## supplementary crystallographic information

### Comment

Spiro compounds are often encountered in many pharmacologically active alkaloids (Cravotto *et al.*, 2001) and fluorene derivatives have been found to have anticonvulsant activity (Vanvakides *et al.*, 2004). In addition, fluorene derivatives, including polyfluorenes and oligofluorenes, have been studied extensively in recent years because they are very promising candidates for blue light-emitting materials in organic light-emitting devices (Muller *et al.*, 2003), organic phototransistors (Saragi *et al.*, 2004), nonlinear optics (Kim *et al.*, 1998) and photochromic materials (Chun *et al.*, 2003). Furan derivatives and annulated furan derivatives occur widely in nature and, along with their unnatural analogs, have been shown to have a wide range of biological activity as well as being important precursors for the synthesis of natural products (Greve & Friedrichsen, 2000). In view of these important properties, the crystal structure of the title compound, (I), has been determined.

in (I, Fig. 1) the C4-C5 and C4-C16 bond distances of the fluorene moiety are almost identical to the values reported in another spiro-linked system (Feng *et al.*, 2004).

The benzene ring is planar with the largest displacement observed being -0.014 (1) Å for atom C22. The deviations of the atoms Cl1 and Cl2 from the least-squares plane of the phenyl rings are -0.114 (1) and 0.015 (1) Å, respectively.

The five membered fluorene moiety adopts an envelope conformation (flap atom C4) with a pseudo-twofold axis passing through the C4-C5 bond. The puckering parameters (Cremer & Pople, 1975) and the lowest displacement asymmetry parameters (Nardelli, 1983) for this ring are q_2_ = 0.107 (2) Å, φ = 355.0 (1)° and Δ_S_(C4) is 1.3 (1)°. The tetrahydrofuran ring also adopts an envelope conformation (flap atom C2) with a pseudo-twofold axis passing through the C2-C3 bond. The puckering parameters (Cremer & Pople, 1975) and the lowest displacement asymmetry parameters (Nardelli, 1983) for this ring are q_2_ = 0.388 (2) Å, φ = 252.1 (2)° and Δ_S_(C2) is 2.1 (2)°.

Carbonyl atom O3 acts as a intermolecuar bifurcated acceptor with both C2 and C6 (Table 1 and Fig. 2) from a symmetry-related molecule to form centrosymmetric hydrogen bonded dimers with cyclic *R*_2_^2^(16) and *R*_2_^2^(8) (Bernstein, *et al.*, 1995) ring systems, respectively. The structure is further stabilized by C—H···π interactions involing rings C26- H26C···*Cg*1 (*Cg*1 is the centroid of the C11—C16 ring).

### Experimental

To a stirred mixture of 9-(2,4-Dichloro-benzylidene)-9*H*-fluorene (1.0 mmol), ethylacetoacetate (1.0 mmol) and NaHCO_3_ (3.0 mmol) in acetonitrile (10 ml), ceric ammonium nitrate (2.5 mmol) dissolved in acetonitrile (5 ml) was added dropwise at 0 ° under N_2_. The reaction mixture was stirred until completion of the reaction as monitored by TLC. Water was added to the mixture and the product was extracted with ethyl acetate (2 × 20 ml) and then dried over anhydrous Na_2_SO_4_. Removal of the solvent under reduced pressure gave a crude product, which was purified by column chromatography on silica gel, with ethyl acetate-hexane (4:6) as eluent to afford a pure product in 79% yield. Single crystals of the title compound suitable for X-ray diffraction were obtained by slow evaporation of a solution in ethylacetate.

### Refinement

All H atoms were positioned geometrically, with O—H = 0.82 and C—H = 0.93–0.98 Å and constrained to ride on their parent atoms, with *U*_iso_(H) = *xU*_eq_(C, N), where *x* = 1.5 for methyl H and *x* = 1.2 for all H atoms.

### Crystal data

**Table:**

C_26_H_22_Cl_2_O_4_	*F*(000) = 1952
*M**_r_* = 469.34	*D*_x_ = 1.379 Mg m^−^^3^
Monoclinic, *C*2/*c*	Mo *K*α radiation, λ = 0.71073 Å
Hall symbol: -C 2yc	Cell parameters from 7176 reflections
*a* = 28.6811 (13) Å	θ = 2.5–25°
*b* = 9.0600 (4) Å	µ = 0.32 mm^−^^1^
*c* = 17.4074 (8) Å	*T* = 293 K
β = 92.072 (3)°	Prismatic, yellow
*V* = 4520.4 (4) Å^3^	0.25 × 0.20 × 0.20 mm
*Z* = 8	

### Data collection

**Table:**

Bruker Kappa APEXII CCD diffractometer	5338 independent reflections
Radiation source: fine-focus sealed tube	3663 reflections with *I* > 2σ(*I*)
graphite	*R*_int_ = 0.056
ω and φ scans	θ_max_ = 27.8°, θ_min_ = 1.4°
Absorption correction: multi-scan (*SADABS*; Bruker 2004)	*h* = −36→37
*T*_min_ = 0.916, *T*_max_ = 0.938	*k* = −11→11
22504 measured reflections	*l* = −21→22

### Refinement

**Table:**

Refinement on *F*^2^	Primary atom site location: structure-invariant direct methods
Least-squares matrix: full	Secondary atom site location: difference Fourier map
*R*[*F*^2^ > 2σ(*F*^2^)] = 0.048	Hydrogen site location: inferred from neighbouring sites
*wR*(*F*^2^) = 0.151	H-atom parameters constrained
*S* = 1.04	*w* = 1/[σ^2^(*F*_o_^2^) + (0.0729*P*)^2^ + 2.471*P*] where *P* = (*F*_o_^2^ + 2*F*_c_^2^)/3
5338 reflections	(Δ/σ)_max_ < 0.001
291 parameters	Δρ_max_ = 0.38 e Å^−^^3^
0 restraints	Δρ_min_ = −0.35 e Å^−^^3^

### Special details

**Table:**

Geometry. All e.s.d.'s (except the e.s.d. in the dihedral angle between two l.s. planes) are estimated using the full covariance matrix. The cell e.s.d.'s are taken into account individually in the estimation of e.s.d.'s in distances, angles and torsion angles; correlations between e.s.d.'s in cell parameters are only used when they are defined by crystal symmetry. An approximate (isotropic) treatment of cell e.s.d.'s is used for estimating e.s.d.'s involving l.s. planes.
Refinement. Refinement of *F*^2^ against ALL reflections. The weighted *R*-factor *wR* and goodness of fit *S* are based on *F*^2^, conventional *R*-factors *R* are based on *F*, with *F* set to zero for negative *F*^2^. The threshold expression of *F*^2^ > σ(*F*^2^) is used only for calculating *R*-factors(gt) *etc*. and is not relevant to the choice of reflections for refinement. *R*-factors based on *F*^2^ are statistically about twice as large as those based on *F*, and *R*- factors based on ALL data will be even larger.

### Fractional atomic coordinates and isotropic or equivalent isotropic displacement parameters (Å^2^)

**Table:**

	*x*	*y*	*z*	*U*_iso_*/*U*_eq_	
Cl1	0.06337 (2)	−0.03027 (7)	0.10063 (4)	0.0663 (2)	
Cl2	0.00329 (2)	0.39144 (8)	−0.09056 (4)	0.0652 (2)	
O1	0.21156 (5)	0.19309 (19)	0.21266 (8)	0.0476 (4)	
O2	0.23543 (6)	−0.03879 (18)	0.17193 (10)	0.0550 (4)	
H2	0.2506	−0.0562	0.2117	0.082*	
O3	0.25673 (6)	0.04006 (18)	−0.01270 (10)	0.0540 (4)	
O4	0.18812 (5)	−0.06733 (17)	0.00380 (9)	0.0455 (4)	
C1	0.23639 (7)	0.1143 (2)	0.15672 (13)	0.0411 (5)	
C2	0.20777 (6)	0.1382 (2)	0.08249 (11)	0.0331 (4)	
H2A	0.2135	0.2389	0.0645	0.040*	
C3	0.15806 (6)	0.1308 (2)	0.10976 (11)	0.0301 (4)	
H3	0.1508	0.0267	0.1186	0.036*	
C4	0.16289 (7)	0.2080 (2)	0.19036 (12)	0.0349 (4)	
C5	0.14777 (8)	0.3679 (2)	0.19083 (12)	0.0401 (5)	
C6	0.16812 (10)	0.4898 (2)	0.15801 (13)	0.0536 (6)	
H6	0.1963	0.4821	0.1336	0.064*	
C7	0.14497 (14)	0.6249 (3)	0.16271 (16)	0.0709 (9)	
H7	0.1579	0.7085	0.1409	0.085*	
C8	0.10342 (13)	0.6362 (3)	0.19909 (17)	0.0722 (9)	
H8	0.0883	0.7269	0.2005	0.087*	
C9	0.08401 (11)	0.5172 (3)	0.23298 (16)	0.0628 (7)	
H9	0.0561	0.5263	0.2581	0.075*	
C10	0.10643 (8)	0.3822 (2)	0.22951 (12)	0.0438 (5)	
C11	0.09519 (8)	0.2392 (3)	0.26364 (13)	0.0442 (5)	
C12	0.05912 (10)	0.1956 (4)	0.30938 (16)	0.0650 (7)	
H12	0.0350	0.2601	0.3201	0.078*	
C13	0.05993 (12)	0.0536 (4)	0.33873 (18)	0.0767 (9)	
H13	0.0359	0.0223	0.3693	0.092*	
C14	0.09547 (12)	−0.0415 (3)	0.32353 (16)	0.0690 (8)	
H14	0.0959	−0.1349	0.3457	0.083*	
C15	0.13054 (9)	−0.0010 (3)	0.27593 (14)	0.0505 (6)	
H15	0.1540	−0.0672	0.2639	0.061*	
C16	0.13012 (7)	0.1400 (2)	0.24653 (11)	0.0378 (5)	
C17	0.12017 (6)	0.1925 (2)	0.05700 (11)	0.0300 (4)	
C18	0.12700 (7)	0.3165 (2)	0.01180 (12)	0.0369 (5)	
H18	0.1564	0.3597	0.0125	0.044*	
C19	0.09199 (8)	0.3780 (2)	−0.03393 (13)	0.0431 (5)	
H19	0.0978	0.4604	−0.0640	0.052*	
C20	0.04839 (7)	0.3160 (2)	−0.03454 (12)	0.0405 (5)	
C21	0.03967 (7)	0.1925 (2)	0.00810 (12)	0.0413 (5)	
H21	0.0101	0.1506	0.0074	0.050*	
C22	0.07576 (7)	0.1313 (2)	0.05219 (12)	0.0355 (4)	
C23	0.28555 (8)	0.1735 (3)	0.15764 (15)	0.0597 (7)	
H23A	0.3002	0.1568	0.2073	0.089*	
H23B	0.2848	0.2774	0.1471	0.089*	
H23C	0.3029	0.1239	0.1192	0.089*	
C24	0.22049 (7)	0.0327 (2)	0.01982 (12)	0.0356 (4)	
C25	0.19678 (9)	−0.1675 (3)	−0.05956 (14)	0.0541 (6)	
H25A	0.2275	−0.2115	−0.0531	0.065*	
H25B	0.1952	−0.1145	−0.1080	0.065*	
C26	0.16055 (11)	−0.2826 (3)	−0.05889 (18)	0.0685 (8)	
H26A	0.1637	−0.3381	−0.0120	0.103*	
H26B	0.1641	−0.3474	−0.1019	0.103*	
H26C	0.1303	−0.2373	−0.0623	0.103*	

### Atomic displacement parameters (Å^2^)

**Table:**

	*U*^11^	*U*^22^	*U*^33^	*U*^12^	*U*^13^	*U*^23^
Cl1	0.0460 (4)	0.0596 (4)	0.0915 (5)	−0.0247 (3)	−0.0215 (3)	0.0354 (3)
Cl2	0.0504 (4)	0.0691 (4)	0.0743 (5)	0.0082 (3)	−0.0234 (3)	0.0176 (3)
O1	0.0330 (8)	0.0669 (10)	0.0423 (9)	−0.0021 (7)	−0.0079 (7)	−0.0085 (7)
O2	0.0522 (10)	0.0538 (10)	0.0579 (10)	0.0060 (8)	−0.0121 (8)	0.0140 (8)
O3	0.0441 (10)	0.0515 (10)	0.0677 (11)	−0.0063 (7)	0.0194 (8)	−0.0078 (8)
O4	0.0390 (8)	0.0434 (8)	0.0540 (9)	−0.0071 (7)	−0.0003 (7)	−0.0137 (7)
C1	0.0308 (11)	0.0483 (12)	0.0439 (12)	−0.0006 (9)	−0.0042 (9)	0.0008 (9)
C2	0.0267 (10)	0.0317 (10)	0.0406 (11)	−0.0033 (7)	−0.0025 (8)	0.0009 (8)
C3	0.0268 (10)	0.0263 (9)	0.0368 (10)	−0.0033 (7)	−0.0030 (8)	0.0011 (7)
C4	0.0318 (10)	0.0345 (10)	0.0381 (11)	−0.0020 (8)	−0.0041 (8)	0.0015 (8)
C5	0.0517 (13)	0.0346 (11)	0.0333 (10)	−0.0043 (9)	−0.0074 (9)	−0.0035 (8)
C6	0.0795 (18)	0.0389 (12)	0.0423 (13)	−0.0168 (12)	−0.0022 (12)	−0.0051 (10)
C7	0.130 (3)	0.0322 (13)	0.0493 (15)	−0.0157 (15)	−0.0189 (17)	0.0005 (11)
C8	0.110 (3)	0.0433 (15)	0.0613 (17)	0.0191 (15)	−0.0230 (18)	−0.0063 (13)
C9	0.0715 (18)	0.0554 (16)	0.0603 (16)	0.0205 (13)	−0.0138 (14)	−0.0078 (13)
C10	0.0489 (13)	0.0430 (12)	0.0387 (11)	0.0062 (10)	−0.0081 (10)	−0.0037 (9)
C11	0.0400 (12)	0.0532 (13)	0.0392 (12)	−0.0001 (10)	−0.0028 (10)	−0.0026 (10)
C12	0.0506 (15)	0.085 (2)	0.0606 (17)	0.0010 (14)	0.0145 (13)	−0.0025 (15)
C13	0.071 (2)	0.092 (2)	0.069 (2)	−0.0250 (18)	0.0225 (16)	0.0101 (17)
C14	0.089 (2)	0.0612 (17)	0.0574 (17)	−0.0222 (16)	0.0069 (16)	0.0162 (13)
C15	0.0611 (16)	0.0435 (13)	0.0467 (13)	−0.0029 (11)	−0.0020 (12)	0.0064 (10)
C16	0.0388 (11)	0.0412 (11)	0.0330 (10)	−0.0042 (9)	−0.0033 (9)	0.0019 (8)
C17	0.0281 (9)	0.0277 (9)	0.0340 (10)	−0.0013 (7)	−0.0029 (8)	−0.0039 (7)
C18	0.0336 (11)	0.0337 (10)	0.0430 (11)	−0.0071 (8)	−0.0048 (9)	0.0018 (9)
C19	0.0471 (13)	0.0347 (11)	0.0469 (12)	−0.0054 (9)	−0.0079 (10)	0.0077 (9)
C20	0.0370 (11)	0.0400 (11)	0.0434 (12)	0.0052 (9)	−0.0107 (9)	−0.0004 (9)
C21	0.0309 (11)	0.0431 (11)	0.0493 (13)	−0.0056 (9)	−0.0070 (9)	−0.0004 (10)
C22	0.0317 (10)	0.0322 (10)	0.0424 (11)	−0.0061 (8)	−0.0030 (9)	0.0027 (8)
C23	0.0327 (12)	0.0820 (18)	0.0634 (16)	−0.0086 (12)	−0.0113 (11)	−0.0038 (14)
C24	0.0340 (11)	0.0305 (10)	0.0419 (11)	0.0004 (8)	−0.0020 (9)	0.0041 (8)
C25	0.0615 (16)	0.0469 (13)	0.0534 (14)	0.0042 (12)	−0.0065 (12)	−0.0132 (11)
C26	0.078 (2)	0.0479 (15)	0.0779 (19)	−0.0077 (13)	−0.0150 (16)	−0.0140 (13)

### Geometric parameters (Å, °)

**Table:**

Cl1—C22	1.733 (2)	C11—C16	1.386 (3)
Cl2—C20	1.732 (2)	C11—C12	1.386 (3)
O1—C1	1.420 (3)	C12—C13	1.384 (4)
O1—C4	1.442 (2)	C12—H12	0.9300
O2—C1	1.412 (3)	C13—C14	1.368 (4)
O2—H2	0.8200	C13—H13	0.9300
O3—C24	1.203 (3)	C14—C15	1.376 (4)
O4—C24	1.320 (2)	C14—H14	0.9300
O4—C25	1.456 (3)	C15—C16	1.376 (3)
C1—C23	1.508 (3)	C15—H15	0.9300
C1—C2	1.521 (3)	C17—C22	1.389 (3)
C2—C24	1.506 (3)	C17—C18	1.389 (3)
C2—C3	1.520 (3)	C18—C19	1.377 (3)
C2—H2A	0.9800	C18—H18	0.9300
C3—C17	1.505 (3)	C19—C20	1.371 (3)
C3—C4	1.569 (3)	C19—H19	0.9300
C3—H3	0.9800	C20—C21	1.371 (3)
C4—C16	1.512 (3)	C21—C22	1.382 (3)
C4—C5	1.512 (3)	C21—H21	0.9300
C5—C6	1.383 (3)	C22—Cl1	1.733 (2)
C5—C10	1.391 (3)	C23—H23A	0.9600
C6—C7	1.396 (4)	C23—H23B	0.9600
C6—H6	0.9300	C23—H23C	0.9600
C7—C8	1.373 (4)	C25—C26	1.472 (4)
C7—H7	0.9300	C25—H25A	0.9700
C8—C9	1.358 (4)	C25—H25B	0.9700
C8—H8	0.9300	C26—H26A	0.9600
C9—C10	1.384 (3)	C26—H26B	0.9600
C9—H9	0.9300	C26—H26C	0.9600
C10—C11	1.466 (3)		
			
C1—O1—C4	111.55 (15)	C14—C13—H13	119.4
C1—O2—H2	109.5	C12—C13—H13	119.4
C24—O4—C25	116.72 (17)	C13—C14—C15	121.0 (3)
O2—C1—O1	110.58 (18)	C13—C14—H14	119.5
O2—C1—C23	111.83 (19)	C15—C14—H14	119.5
O1—C1—C23	107.81 (18)	C14—C15—C16	118.4 (3)
O2—C1—C2	106.62 (17)	C14—C15—H15	120.8
O1—C1—C2	104.00 (16)	C16—C15—H15	120.8
C23—C1—C2	115.72 (19)	C15—C16—C11	121.2 (2)
C24—C2—C3	116.91 (16)	C15—C16—C4	128.4 (2)
C24—C2—C1	112.78 (17)	C11—C16—C4	110.23 (18)
C3—C2—C1	102.23 (16)	C22—C17—C18	115.87 (18)
C24—C2—H2A	108.2	C22—C17—C3	121.93 (17)
C3—C2—H2A	108.2	C18—C17—C3	122.18 (17)
C1—C2—H2A	108.2	C19—C18—C17	122.68 (19)
C17—C3—C2	117.24 (16)	C19—C18—H18	118.7
C17—C3—C4	114.73 (15)	C17—C18—H18	118.7
C2—C3—C4	101.89 (15)	C20—C19—C18	119.0 (2)
C17—C3—H3	107.5	C20—C19—H19	120.5
C2—C3—H3	107.5	C18—C19—H19	120.5
C4—C3—H3	107.5	C19—C20—C21	120.98 (19)
O1—C4—C16	113.98 (16)	C19—C20—Cl2	120.33 (17)
O1—C4—C5	111.29 (16)	C21—C20—Cl2	118.68 (16)
C16—C4—C5	101.64 (17)	C20—C21—C22	118.69 (19)
O1—C4—C3	104.61 (15)	C20—C21—H21	120.7
C16—C4—C3	111.10 (16)	C22—C21—H21	120.7
C5—C4—C3	114.56 (16)	C21—C22—C17	122.71 (18)
C6—C5—C10	120.4 (2)	C21—C22—Cl1	116.49 (15)
C6—C5—C4	129.6 (2)	C17—C22—Cl1	120.78 (15)
C10—C5—C4	109.98 (18)	C21—C22—Cl1	116.49 (15)
C5—C6—C7	117.9 (3)	C17—C22—Cl1	120.78 (15)
C5—C6—H6	121.1	C1—C23—H23A	109.5
C7—C6—H6	121.1	C1—C23—H23B	109.5
C8—C7—C6	121.0 (3)	H23A—C23—H23B	109.5
C8—C7—H7	119.5	C1—C23—H23C	109.5
C6—C7—H7	119.5	H23A—C23—H23C	109.5
C9—C8—C7	121.2 (3)	H23B—C23—H23C	109.5
C9—C8—H8	119.4	O3—C24—O4	123.5 (2)
C7—C8—H8	119.4	O3—C24—C2	122.80 (19)
C8—C9—C10	119.0 (3)	O4—C24—C2	113.70 (17)
C8—C9—H9	120.5	O4—C25—C26	107.2 (2)
C10—C9—H9	120.5	O4—C25—H25A	110.3
C9—C10—C5	120.6 (2)	C26—C25—H25A	110.3
C9—C10—C11	130.8 (2)	O4—C25—H25B	110.3
C5—C10—C11	108.50 (19)	C26—C25—H25B	110.3
C16—C11—C12	119.9 (2)	H25A—C25—H25B	108.5
C16—C11—C10	108.35 (19)	C25—C26—H26A	109.5
C12—C11—C10	131.6 (2)	C25—C26—H26B	109.5
C13—C12—C11	118.3 (3)	H26A—C26—H26B	109.5
C13—C12—H12	120.8	C25—C26—H26C	109.5
C11—C12—H12	120.8	H26A—C26—H26C	109.5
C14—C13—C12	121.1 (3)	H26B—C26—H26C	109.5
			
C4—O1—C1—O2	−89.2 (2)	C10—C11—C12—C13	−174.5 (3)
C4—O1—C1—C23	148.26 (19)	C11—C12—C13—C14	0.4 (5)
C4—O1—C1—C2	24.9 (2)	C12—C13—C14—C15	−2.7 (5)
O2—C1—C2—C24	−48.0 (2)	C13—C14—C15—C16	2.7 (4)
O1—C1—C2—C24	−164.86 (16)	C14—C15—C16—C11	−0.5 (3)
C23—C1—C2—C24	77.1 (2)	C14—C15—C16—C4	−176.5 (2)
O2—C1—C2—C3	78.44 (19)	C12—C11—C16—C15	−1.9 (3)
O1—C1—C2—C3	−38.47 (19)	C10—C11—C16—C15	175.3 (2)
C23—C1—C2—C3	−156.49 (19)	C12—C11—C16—C4	174.9 (2)
C24—C2—C3—C17	−73.5 (2)	C10—C11—C16—C4	−8.0 (2)
C1—C2—C3—C17	162.79 (16)	O1—C4—C16—C15	−52.6 (3)
C24—C2—C3—C4	160.35 (16)	C5—C4—C16—C15	−172.4 (2)
C1—C2—C3—C4	36.69 (18)	C3—C4—C16—C15	65.3 (3)
C1—O1—C4—C16	120.28 (19)	O1—C4—C16—C11	131.00 (19)
C1—O1—C4—C5	−125.50 (19)	C5—C4—C16—C11	11.2 (2)
C1—O1—C4—C3	−1.3 (2)	C3—C4—C16—C11	−111.1 (2)
C17—C3—C4—O1	−150.41 (16)	C2—C3—C17—C22	145.93 (19)
C2—C3—C4—O1	−22.69 (18)	C4—C3—C17—C22	−94.6 (2)
C17—C3—C4—C16	86.2 (2)	C2—C3—C17—C18	−35.9 (3)
C2—C3—C4—C16	−146.11 (16)	C4—C3—C17—C18	83.6 (2)
C17—C3—C4—C5	−28.3 (2)	C22—C17—C18—C19	1.4 (3)
C2—C3—C4—C5	99.43 (19)	C3—C17—C18—C19	−176.88 (19)
O1—C4—C5—C6	49.6 (3)	C17—C18—C19—C20	0.7 (3)
C16—C4—C5—C6	171.4 (2)	C18—C19—C20—C21	−1.5 (3)
C3—C4—C5—C6	−68.8 (3)	C18—C19—C20—Cl2	179.31 (17)
O1—C4—C5—C10	−132.24 (18)	C19—C20—C21—C22	0.2 (3)
C16—C4—C5—C10	−10.5 (2)	Cl2—C20—C21—C22	179.34 (16)
C3—C4—C5—C10	109.4 (2)	C20—C21—C22—C17	2.1 (3)
C10—C5—C6—C7	−2.3 (3)	C20—C21—C22—Cl1	−176.59 (17)
C4—C5—C6—C7	175.7 (2)	C20—C21—C22—Cl1	−176.59 (17)
C5—C6—C7—C8	0.3 (4)	C18—C17—C22—C21	−2.8 (3)
C6—C7—C8—C9	1.4 (4)	C3—C17—C22—C21	175.45 (19)
C7—C8—C9—C10	−0.9 (4)	C18—C17—C22—Cl1	175.82 (15)
C8—C9—C10—C5	−1.1 (4)	C3—C17—C22—Cl1	−5.9 (3)
C8—C9—C10—C11	176.4 (2)	C18—C17—C22—Cl1	175.82 (15)
C6—C5—C10—C9	2.7 (3)	C3—C17—C22—Cl1	−5.9 (3)
C4—C5—C10—C9	−175.6 (2)	C25—O4—C24—O3	−3.3 (3)
C6—C5—C10—C11	−175.3 (2)	C25—O4—C24—C2	176.29 (18)
C4—C5—C10—C11	6.4 (2)	C3—C2—C24—O3	170.8 (2)
C9—C10—C11—C16	−176.7 (2)	C1—C2—C24—O3	−71.1 (3)
C5—C10—C11—C16	1.0 (2)	C3—C2—C24—O4	−8.7 (3)
C9—C10—C11—C12	−0.1 (4)	C1—C2—C24—O4	109.3 (2)
C5—C10—C11—C12	177.7 (3)	C24—O4—C25—C26	171.0 (2)
C16—C11—C12—C13	1.9 (4)		

### Hydrogen-bond geometry (Å, °)

**Table:**

*D*—H···*A*	*D*—H	H···*A*	*D*···*A*	*D*—H···*A*
C2—H2A···O3^i^	0.98	2.37	3.331 (3)	167
C6—H6···O3^i^	0.93	2.55	3.393 (3)	151
C3—H3···Cl1	0.98	2.57	3.082 (2)	113
C26—H26C···Cg1^ii^	0.96	2.95	3.556 (1)	122

Symmetry codes: (i) −*x*+1/2, −*y*+1/2, −*z*;  (ii) −*x*+1/2, *y*+1/2, −*z*−1/2.
